# Application of the i-PARIHS framework for enhancing understanding of interactive dissemination to achieve wide-scale improvement in Indigenous primary healthcare

**DOI:** 10.1186/s12961-018-0392-z

**Published:** 2018-11-29

**Authors:** Alison Laycock, Gillian Harvey, Nikki Percival, Frances Cunningham, Jodie Bailie, Veronica Matthews, Kerry Copley, Louise Patel, Ross Bailie

**Affiliations:** 1 Menzies School of Health Research, Charles Darwin University, PO Box 41096, Darwin, Casuarina Northern Territory 0811 Australia; 2 Adelaide Nursing School, The University of Adelaide, North Tce, Adelaide, SA 5000 Australia; 3 The Australian Centre for Public and Population Health Research, University of Technology Sydney, PO Box 123, Broadway, Ultimo, NSW 2007 Australia; 4 The University of Sydney, University Centre for Rural Health, 61 Uralba Street, Lismore, NSW 2480 Australia; 5 Aboriginal Medical Services Alliance Northern Territory, GPO Box 1624, Darwin, Northern Territory 0801 Australia

**Keywords:** Dissemination, interactive, i-PARIHS, integrated knowledge translation, continuous quality improvement, Indigenous health, participatory research, system level, engagement, developmental evaluation

## Abstract

**Background:**

Participatory research approaches improve the use of evidence in policy, programmes and practice. Few studies have addressed ways to scale up participatory research for wider system improvement or the intensity of effort required. We used the integrated Promoting Action on Research Implementation in Health Services (i-PARIHS) framework to analyse implementation of an interactive dissemination process engaging stakeholders with continuous quality improvement (CQI) data from Australian Indigenous primary healthcare centres. This paper reports lessons learnt about scaling knowledge translation research, facilitating engagement at a system level and applying the i-PARIHS framework to a system-level intervention.

**Methods:**

Drawing on a developmental evaluation of our dissemination process, we conducted a post-hoc analysis of data from project records and interviews with 30 stakeholders working in Indigenous health in different roles, organisation types and settings in one Australian jurisdiction and with national participants. Content-analysed data were mapped onto the i-PARIHS framework constructs to examine factors contributing to the success (or otherwise) of the process.

**Results:**

The dissemination process achieved wide reach, with stakeholders using aggregated CQI data to identify system-wide priority evidence–practice gaps, barriers and strategies for improvement across the scope of care. Innovation characteristics influencing success were credible data, online dissemination and recruitment through established networks, research goals aligned with stakeholders’ interest in knowledge-sharing and motivation to improve care, and iterative phases of reporting and feedback. The policy environment and infrastructure for CQI, as well as manager support, influenced participation. Stakeholders who actively facilitated organisational- and local-level engagement were important for connecting others with the data and with the externally located research team. Developmental evaluation was facilitative in that it supported real-time adaptation and tailoring to stakeholders and context.

**Conclusions:**

A participatory research process was successfully implemented at scale without intense facilitation efforts. These findings broaden the notion of facilitation and support the utility of the i-PARIHS framework for planning participatory knowledge translation research at a system level. Researchers planning similar interventions should work through established networks and identify organisational- or local-level facilitators within the research design. Further research exploring facilitation in system-level interventions and the use of interactive dissemination processes in other settings is needed.

**Electronic supplementary material:**

The online version of this article (10.1186/s12961-018-0392-z) contains supplementary material, which is available to authorized users.

## Background

Participatory research approaches improve the use of evidence in policy, programmes and practice [1–3]. There is a growing body of research involving research users as active partners and expert contributors in research [4]. System-wide continuous quality improvement (CQI) approaches are associated with wide-scale improvement in care [5, 6]. However, little is known about how to scale up participatory research and integrate evidence use for wider system improvement and population health impact [7], or the resources required for productive researcher–user collaboration at a system level [7, 8]. These gaps in knowledge provide scope to explore whether (and how) the feedback and interpretation processes used to engage healthcare teams with local audit data [9] can be scaled to target higher-level system change, and the intensity of facilitation effort required.

The integrated Promoting Action on Research Implementation in Health Services (i-PARIHS) framework [10, 11] was designed to facilitate evidence use at the practice level. Integrating four constructs commonly identified in knowledge translation literature (i.e. context, innovation features, individual characteristics and implementation processes), the framework draws on theory about how organisations learn and considers the wider implementation context [12, 13]. These features suggest i-PARIHS has utility for evidence use at higher levels of the health system such as regional and national levels. To our knowledge, i-PARIHS has not been applied as an analytical framework to examine implementation constructs within a system level research project.

### Indigenous primary healthcare (PHC) and CQI

Aboriginal and Torres Strait Islander Australians (hereafter respectfully referred to as Indigenous), experience poorer health outcomes and shorter life expectancy compared with the general Australian population [14, 15]. The causes of these disparities are complex, including colonisation and associated trauma, socioeconomic inequality and racism. Access to high quality PHC is an essential part of efforts to close this equity gap. Among other features, PHC should be client centred and culturally safe, based on the best available evidence and consider influences operating at different levels, including professional roles and skill mix, organisation-related factors and the wider policy context [16].

Indigenous Australians access PHC through community-controlled and government-managed services specifically designed to meet their needs as well as through private general practices [17]. A national CQI project, the Audit and Best Practice in Chronic Disease (ABCD) National Research Partnership (2010–2014), built on several years of CQI research and implementation in Indigenous PHC to develop and scale a systems approach to improving PHC quality [18–21]. Throughout this progression, participatory approaches were critical for upholding Indigenous values as expressed in national statements on research and cultural respect [22]. Participatory approaches were also used for achieving consensus as evidence-based CQI tools were developed and for encouraging the uptake of CQI [18, 23]. PHC centres used the evidence-based best-practice clinical audit and systems assessment tools [24] to assess and reflect on system performance and to tailor improvement interventions to community and service contexts. Over 175 Indigenous PHC centres using these CQI processes voluntarily provided de-identified clinical audit and systems assessment data to the ABCD National Research Partnership for analysis. Sustained CQI efforts achieved improved delivery of evidence-based PHC and ultimately health outcomes [6, 18, 25, 26]. Though there were many areas of care in which PHC centres were doing well, there were persistent gaps between evidence and practice and wide variation in performance between PHC centres [25, 27–29].

### Scaling up participatory CQI research and evidence use

**Fig. 1:**
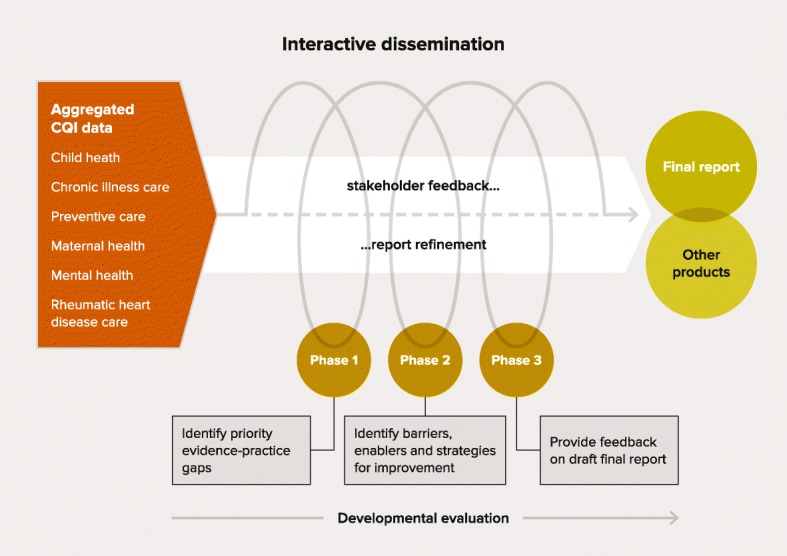
Interactive dissemination process used in the ESP Project

Drawing on this CQI research in Indigenous PHC [19, 20, 23] and principles of knowledge co-production [30, 31], our team designed a large-scale project titled ‘Engaging Stakeholders in Identifying Priority Evidence-Practice Gaps, Barriers and Strategies for Improvement’ (ESP) [32] (Box 1). The ESP project brought together participatory research and dissemination in a process we described as ‘interactive dissemination’ [32]. Stakeholders working at different levels of the health system were engaged to interpret aggregated CQI data from Indigenous PHC centres to (1) identify priority gaps in key areas of care provision and (2) reflect on the key barriers and enablers as well as to suggest strategies for improvement. A concurrent developmental evaluation used an embedded researcher-evaluator to inform continuous project refinement [33] (Fig. 1).

This paper applies the i-PARIHS framework to analyse the implementation of the ESP project. It aims to identify lessons for teams scaling up participatory knowledge translation research and for facilitating engagement at a system level. Our study also examines whether the i-PARIHS framework can be applied to a system-level intervention.

## Methods

We drew on the developmental evaluation [34] of the ESP project, which aimed to explore facilitators and barriers to stakeholder engagement and use of project findings, inform ongoing project refinement and implementation, and appraise the interactive dissemination process. The evaluation design was integrated into the iterative design of the ESP project. The developmental evaluator role was undertaken by a member of the project team that comprised one Indigenous and three non-Indigenous researchers. Document analysis, surveys and stakeholder interviews were used to collect evaluation data between 2014 and 2017. The embedded evaluator led the team in using these data to systematically assess implementation, plan responses and adjust the ESP reports and processes in real-time [35]. Evaluation methods are described elsewhere [33].

### Project records

Stakeholder responses to survey items about the ESP reports and surveys, administrative records of team communications and meetings, ESP reports, and a timeline of key project decisions and adaptations were reviewed to inform this study. The summary of results and list of ESP team processes, responses and adaptations presented in Box 2 are based on a review of this material.

### Data from stakeholder interviews

The developmental evaluation involved semi-structured interviews with 30 stakeholders from both government-managed and Indigenous community-controlled services (*n* = 17), research/teaching institutions (*n* = 7) and support organisations, including regional health networks and non-government organisations representing member services (e.g. Indigenous community-controlled health services) (*n* = 6). Ten clinicians, 6 academics, 5 CQI practitioners, 5 managers and 4 policy-makers were interviewed by the developmental evaluator (and lead author (AL)) between mid-2015 and early 2017. Many interviewees had worked for 10 or more years in Indigenous PHC. Interviews of between 23 and 75 minutes in length were conducted face-to-face, by Skype or telephone. All were audio-recorded and transcribed.

The interview questions explored stakeholders’ motivation, opinions of ESP methods, factors influencing participation, ideas for improving engagement and use of the ESP reports. Data were deductively coded into categories reflecting common themes in knowledge translation theory [13] using NVivo10 analytic software [36]. Data were then inductively coded to identify subcategories, patterns and themes.

### The framework for analysis: i-PARIHS

We identified the i-PARIHS framework post-hoc as a potentially useful analytical aid to provide insights into the characteristics of, and interrelationships between, (1) the ESP design and implementation, (2) stakeholder engagement, (3) influences in the Indigenous PHC environment and (4) refinements made during implementation. The PARiHS framework [37, 38] has been previously identified as an accessible and flexible implementation framework for use in Indigenous Australian PHC services and programmes [39]. The PARiHS framework has been revised by its developers to place greater emphasis on facilitation [40] as the process that activates implementation of the evidence or innovation with intended recipients in their contextual settings [13] (Box 3). Renamed as i-PARIHS, the framework defines a facilitation process as “*a set of strategies and actions to enable implementation*” ([13], p. 44). This definition aligns with the way we perceived the ESP process.

### Defining successful implementation of the ESP project

Consistent with the goals of the ESP project and the objectives of the developmental evaluation, evidence of successful implementation was expected to include:

- Wide distribution of ESP reports among Indigenous PHC stakeholders in diverse roles and settings
- Engagement of diverse Indigenous PHC stakeholders in interpreting aggregated CQI data
- Identification of priority evidence–practice gaps, barriers, enablers and strategies for improvement in child health, chronic illness care, preventive health, maternal health, mental health, and rheumatic heart disease care
- Reports of findings regarded by stakeholders as accessible, useful and useable for system-level improvement of PHC
- Application of generated evidence into practice and/or policy

### Data analysis

Interview data previously coded into categories were mapped onto the framework constructs [13] of ‘evidence/innovation characteristics’, ‘recipients’, ‘inner context (local)’, ‘inner context (organisation)’ and ‘outer context’. Consistent with the positioning of the ‘facilitation’ construct within the i-PARIHS framework [10], data within each category were then coded according to what the ESP process focused on (e.g. recipient construct: motivation, time and resources) and how the ESP processes acted on these elements (e.g. recipient construct: consensus building, boundary spanning) [11]. These data were synthesised with information gathered from reviewing project records to inform the study findings.

Table 1 defines the constructs of the i-PARIHS framework and how comparable ESP constructs were defined for the purpose of analysis.

**Table 1:** i-PARIHS constructs and how ESP constructs were defined for analysis

i-PARIHS constructs and definitions	Comparable ESP constructs and definitions
Fac^*n*^ = Facilitation	ESP implementation processes
• The active element that assesses, aligns and integrates the innovation, recipients and context • Uses action-learning techniques to enable adoption of new knowledge into practice • Enables and encourages teams and individuals to reflect and think in a systematic way and to embrace continuous improvement of their practice based on best available evidence • Commonly involves improvement approaches underpinned by project management [11]	• Used networks, built on relationships and used snowballing recruitment to engage stakeholders in data interpretation • Distributed aggregated CQI data and cumulative findings among stakeholders using phased reports • Encouraged teams and individuals to engage with the data, reflect on their practice, systems and context, and think about how to use this evidence to improve care • Gathered group and individual input through phase online surveys culminating in final reports • Used iterative processes based on a CQI approach • Used concurrent developmental evaluation to gather evaluative feedback and put learning into practice, adapt reports and processes to support engagement and use of collaboratively produced evidence
*I* = Innovation	The ESP Innovation
The focus or content of the implementation effort	Used aggregated CQI data from 175 Indigenous PHC centres in 5 jurisdictions to: • Identify priority evidence-practice gaps, barriers/enablers operating at different health system levels and strategies for improvement in key areas of clinical care • Develop accessible, useful and usable reports Used interactive dissemination processes to: • Share explicit evidence and capture stakeholder knowledge to co-produce evidence for improving care • Distribute reports and administer surveys online • Collect and analyse survey responses and prepare reports of ESP findings • Repeat the process using aggregated CQI data in different areas of clinical care
*R* = Recipients (individual, collective)	ESP stakeholders
Staff, support services and patients involved in and affected by implementation and how they respond to the changes required to implement the innovation	Individuals and teams involved in Indigenous PHC and how they responded to the requirements of participating in the ESP project. Stakeholders included: • Indigenous health practitioners, nurses, midwives, doctors, including medical specialists, allied health professionals • CQI practitioners • Middle and senior managers • Policy officers, health board members • Researchers/academics and others
*C* = Context (inner, outer)	ESP Context (inner, outer)
Contextual factors, their potential impact on implementation and how best to handle them	*Inner context: Local* Indigenous PHC centres with diverse: • Range of services, population size • Settings (urban, rural, remote) • Governance arrangements • Infrastructure, resources, staffing • CQI approaches and continuity, ABCD history and policy support [6] *Inner context: organisational* PHC services and centres, support organisations, research institutions, universities, government departments *Outer context* Government policy commitment and funding initiatives addressing health inequity for Indigenous people, CQI research networks, national policy environment for CQI

*ABCD* Audit and Best practice for Chronic Disease, *CQI* continuous quality improvement, *PHC* primary health care, *ESP* Engaging Stakeholders in Identifying Priority Evidence-Practice Gaps, Barriers and Strategies for Improvement

## Results

Successful implementation of the ESP project, as defined above, was achieved. Specifically, initial recipients of phase 1 aggregated CQI data reports were child health (*n* = 98), chronic illness care (*n* = 165), preventive health (*n* = 151), maternal health (*n* = 228), mental health (*n* = 251), and rheumatic heart disease care (*n* = 125). The reach achieved through snowballing dissemination could not be measured; however, approximate numbers of individuals participating in surveys were recorded as: child health (*n* = 134), chronic illness care (*n* = 390), preventive health (*n* = 152), maternal health (*n* = 181), mental health (*n* = 64), and rheumatic heart disease care (*n* = 118) (Table 2). The iterative reporting and feedback processes were effective in engaging diverse Indigenous PHC stakeholders to interpret aggregated CQI data, with priority evidence–practice gaps, improvement barriers, enablers and strategies identified in each area of care. Responses from individuals and groups represented a range of roles and organisation types and included Indigenous respondents (Table 3). The reports and processes were refined as the ESP project progressed and the findings were used in various ways.

**Table 2:** Number of survey responses by ESP process, individual (Ind) and group (Gr) responses, 2014–2017

ESP process	Phase 1 responses			Phase 2 responses			Phase 3 responses			Final draft report		
	Ind	Gr	Total (*n*)	Ind	Gr	Total (*n*)	Ind	Gr	Total (*n*)	Ind	Gr	Total (*n*)
Child health	17	3	38	26	3	47	11	1	18	10	3	31
Chronic illness care	45	10	202	11	4	65	15	3	73	17	6	50
Maternal health	27	6	112	10	3	60	Merged into Phase 2			4	1	9
Preventive health	15	4	77	3	4	70	Merged into Phase 2			5	–	5
Mental health	12	1	14	22	3	50	Merged into Phase 2			–	–	–
Rheumatic heart disease	17	4	50	5	4	68	Merged into Phase 2			–	–	–

Note: When there were less than 5 respondents during the final phase of feedback respondent information is not shown. Some groups indicated large numbers (e.g. 30, > 100, 300 people). For the purpose of estimating the numbers who provided actual input we have used a figure of 20 individuals for groups reported to be larger than 20. The estimated number of people providing input may therefore be conservative. In later cycles, phases 2 and 3 were merged, based on stakeholder feedback about the process

**Table 3:** Number of survey responses by ESP process, roles and organisation type, and percentage of Indigenous respondents, 2014–2017

ESP process	Child health	Chronic illness care	Maternal health	Preventive health	Mental health	Rheumatic heart disease
Total number of survey responses by roles						
Nurse, Midwife	16	32	30	11	9	8
Middle Manager	6	13	3	5	4	2
Doctor, Medical specialist	16	46	18	5	7	12
Executive Manager	1	17	4	7	2	2
CQI practitioner	13	20	3	4	–	3
Board member	1	3	1	1	–	–
Policy officer	5	9	1	2	–	1
Indigenous health practitioner	4	9	9	7	9	6
Academic	14	16	10	2	3	5
Other	16	17	7	9	8	8
Number of survey responses (individual or group) by organisation type						
Community controlled health centre	13	16	19	9	14	11
Community controlled peak body	7	8	–	2	–	1
Government health centre	9	23	12	5	4	5
Government health department	18	39	13	6	4	11
Medicare local network	3	2	7	2	5	3
General Practice	–	6	3	–	–	–
University/Research organisation	15	15	13	3	4	5
Other	15	21	2	5	7	2
Respondents identifying as Indigenous, n (%)						
	14 (10%)	61 (15%)	95 (52%)	60 (39%)	20 (31%)	57 (48%)

Note: Numbers may not tally with total number of respondents as respondents were able to select multiple answers. An individual may have provided responses across phases within each area of care

These results have been examined using the constructs of the i-PARIHS framework to identify the key factors that supported and constrained success. Interdependencies were observed between the facilitation, innovation, recipient and context constructs. We have described the findings according to the predominant construct. Example stakeholder quotes aligned with i-PARIHS constructs are provided in Additional file 1, with some included in the text.

### Facilitation

Facilitation is perceived within the i-PARIHS framework as an active process involving facilitators and facilitation processes. Our research team had limited, episodic contact with stakeholders using mainly online communication. Emailing reports and survey links to contacts in CQI research and Indigenous PHC and encouraging distribution through their networks was an effective recruitment strategy. Interviewees and team members attributed this partly to the long history of collaboration and trust established through the ABCD National Research Partnership. Interaction was fostered through the phased processes of reporting and stakeholder input, as reflected in the following interviewee comment:

“*I like that – ‘This is what you’ve said, we’ve taken that on board, this is the next step. What can we do about it?’ I think that’s really powerful to acknowledge the consultation and to reassure people that their voices have been heard.*” (Academic 3)

Interviewees observed that the ESP project was being adapted in response to feedback. For example, when the phase surveys identifying improvement barriers and strategies were merged to address reported barriers to survey completion, interviewees referred to a greater sense of completion, quicker results and more logical flow, “*because when people think of barriers, they just naturally think of solutions at the same time*” (CQI Practitioner 4). Changes in the presentation of the reports were also noted:

“*As the succession of the topics has come out, I think we’ve seen an improvement in the presentation of the data and even in … the issues you are canvassing*” (Manager 2).

The developmental evaluation facilitated these changes through the team processes of reflection and inquiry that continuously appraised the fit between the ESP implementation and stakeholders’ roles, practice and work contexts, considered options, and made refinements in real-time. This required openness to change and skills to respond to stakeholder feedback (e.g. changing data visualisation, developing plain language summaries), with positive outcomes.

“*It has been necessary for the ESP team to be flexible to accommodate the way our external experts want to have input, and their capacity to have input. … This flexibility means we gain greater engagement with stakeholders overall.*” (ESP team member)

Interviewees commonly spoke about the shortcomings of online dissemination as a strategy for encouraging participatory interpretation of data. However, the number of group survey responses (Table 2) and interview feedback indicated that people facilitated these processes in their work settings. Those in dedicated CQI roles appeared particularly important for ESP implementation. In addition to facilitating group survey responses, they promoted awareness, disseminated reports, sent reminders about surveys, arranged presentations at CQI forums and used ESP reports in CQI activities and training. Several interviewees perceived these roles as key to broader engagement, not least because CQI practitioners had facilitation expertise and a mandate to bring people together for CQI purposes.

In summary, online dissemination, use of professional networks and positive perceptions of research quality extended ESP reach, while participatory research cycles enabled exchange to occur between the research team and stakeholders. The iterative design and concurrent developmental evaluation enabled the dissemination process to be sufficiently interactive and responsive to feedback to sustain engagement despite the team’s limited interpersonal contact with stakeholders. Those in CQI and other leadership roles offset the team’s distant location by facilitating engagement at regional, organisational and local levels. Team facilitation and developmental evaluation processes, responses and adaptations are listed in Box 2.

### The innovation

ESP data were derived from the use of CQI processes familiar to most interviewees and were generally perceived to be trustworthy. Interviewees considered the project novel for presenting data from Indigenous community controlled and government-managed health services together, for enacting a scaled-up CQI cycle using data aggregated at a whole-of-system level (rather than local data) and for seeking wide input.

“*The ESP Project, I could see what it was building on. ... That was the key point of difference for me. … I think the multi-stage process of presenting the evidence and getting the feedback and then presenting the feedback and more evidence and then asking about barriers and enablers* – *that iterative process of* [stakeholder] *involvement is unique.*” (Academic 3)

The ESP design was adapted from a systematic process developed by French et al. [41] to link interventions to modifiable barriers to address evidence–practice gaps. Innovations were made to an implementation tool based on the Theoretical Domains Framework [42, 43], enabling surveys to capture stakeholder knowledge about barriers and enablers operating at health centre and wider system levels [20, 44, 45]. Online report distribution and snowballing recruitment enabled wide reach using modest resources. Survey respondents collectively identified between five and seven priority evidence–practice gaps, four to nine key barriers/enablers, and a range of improvement strategies for each of the areas of care [46–51]. These processes also identified common priority gaps and improvement barriers across areas of care.

Some interviewees in policy and regional roles believed that, while they could comment on strategic barriers, the surveys were “*geared for people working in clinics*” (Policy officer 2). Conversely, a few clinicians thought presenting nationally aggregated CQI data made the process “*a bit remote from the clinical interface*” (Clinician 7), as they were interested primarily in their local-level data.

Early feedback indicated that long reports and ‘academic’ language were discouraging wide participation. This critical feedback resulted in the development of plain language summaries, the presentation of ESP reports in a 1:3:25 format (1-page key messages, 3-page summary, 25-page detailed report) and separate data supplements. The reports included an explanation (with audio-visual link) of how to interpret the graphs, and diagrams were added to support text where deemed necessary. Refinements to report presentation were ongoing (Box 2). Despite the changes, some stakeholders considered the ESP reports too detailed to engage time-poor clinicians and middle managers, reinforcing the value of the summaries:

“*People will read the main messages, but they are unlikely to get beyond that. … it’s beyond most people’s capacity to understand them and to have the time to think about them.*” (Clinician 6)

Repetition and frequency of project processes and long surveys also drew negative comments. As a result, surveys were modified and shortened, timeframes were extended and, as explained above, two project phases were merged. Overall, the surveys were perceived to be user-friendly, as they invited individual or group input and provided opportunities to reflect on practice and systems, share experiences and convey perspectives. A strategy of capturing tacit knowledge was generally regarded as respectful and affirming, “*it’s respecting those practitioners, valuing what they have. And that’s not* [often] *done well… making them feel that they can be part of improving things*” (Clinician 5).

The ESP project findings on evidence–practice gaps and barriers to improvement concurred with many interviewees’ experiences of working in Indigenous PHC. They reportedly prompted reflection on system failures, CQI achievements, and the policy changes required to support more holistic and culturally appropriate care. Stakeholders spoke about the benefits that scale and diversity of input brought to the findings.

“*The ESP has provided another layer of information that’s stimulated thinking and discussion, that’s brought in knowledge and expertise and experience from a broad group. So, it’s really enriched the work that we’ve done*.” (CQI Practitioner 1)

Interviewees perceived the ESP findings as useful for influencing change at different system levels and supporting a systems approach. For example:

“*At the micro level it can just start conversations with people individually and gives people permission to talk about* [a priority] *and to raise it as an issue. And on a macro level, it provides this large scale, very hard to argue with, evidence for why action is needed and support from the wider health system, government funders is needed in terms of resource allocation*.” (Academic 3)

It was reported that ESP project reports were used for informing planning and policy, supporting best practice and reflection, capacity strengthening activities, and for developing new research ideas and grants. This is the topic of a separate publication [52].

In summary, the ESP design and processes were successful in distributing data and co-producing knowledge to identify priority evidence–practice gaps, barriers/enablers and barrier-driven strategies for improvement. The ESP goals, evidence source, degree of novelty and dissemination process stimulated and sustained stakeholder engagement. Building evaluation into the project design and repeating the process using different sets of aggregated CQI data increased opportunities to refine the reports and processes in response to critical feedback from stakeholders, to better fit their needs and implementation contexts.

### Recipients

The number of people providing survey responses varied between ESP cycles and phases in different areas of care (Table 2). The numbers indicate successful snowballing recruitment in earlier cycles for child health and chronic illness care, reducing in later cycles. Respondents represented a broad range of roles and organisations (Table 3).

Across roles and settings, a commitment to improving Indigenous health outcomes was reported as the primary motivation for participating and for using the reports of ESP findings. Access to practice-relevant evidence was linked with improving care and it was important to understand how participation in the project could assist practice.

“*I’m invested in it and believing in data driven health improvement and seeing the value of that*.” (CQI Practitioner 3)

“*If I know it is going to be of some benefit and relevant to me, and I can use it, then I’m more inclined to spend the extra amount of time on it*.” (CQI Practitioner 5)

Another commonly reported motivator was a desire to influence practice and policy. Reasons given for reading reports included interest in benchmarking local data with national- and jurisdiction-level data, reading others’ perspectives, knowing the extent to which findings reflected their own experiences, learning lessons for evaluating CQI implementation, considering whether suggested strategies could be adapted for local use and seeing evidence of personal input. Some said that receiving reports from their manager, or a respected research leader, influenced their decision to participate. Most interviewees had sent the documents on to colleagues.

The majority of respondents were experienced in using CQI processes and were open to using data as a catalyst for consensus-building and for focusing on strategies for better performance. Many people participated in group survey responses (Table 2). Indigenous stakeholders mostly contributed in groups. The perceived value of group input was noted, with interviewees referring to the benefits of discussing data, sharing views, learning from each other and generating ideas.

“*Clinicians are likely to generate a lot more ideas and will have a lot more thoughts if engaged in discussion rather than sitting there individually responding to questions.*” (Manager 1)

Some groups drew on familiar CQI techniques, including systems assessment processes [24]. These consensus processes were considered useful by a group who found the intent of some survey questions unclear.

“*There was a lot of discussion about concepts, what exactly the questions were asking – there were slight differences of opinion and … our experiences are different – and coming up with a consensus about how we would respond.*” (Clinician 3)

Many interviewees spoke about the learning opportunities the project offered. Perceived barriers to engagement were unfamiliarity with academic-style language and lack of confidence in data interpretation skills. Inability to relate the project to routine work and scepticism that individual input could influence policy were also barriers. People in academic roles tended to seek more comprehensive and detailed reports and, together with clinicians, middle managers and policy practitioners, valued the plain language summaries with key messages. Many interviewees reported intended or actual use of the findings in their professional practice. However, it was not possible to measure impact on services or outcomes as part of the evaluation.

Some professional groups were key to wider dissemination and engagement, including ABCD National Research Partnership network members, managers who were CQI champions within their organisations, the clinical experts who collaborated in data analysis (e.g. a mental health specialist), and CQI practitioners. Several people had long-term involvement with the CQI research programme and had worked collaboratively with ESP team members.

In summary, interviewees shared a commitment to improving health outcomes and were motivated to access the data and the shared knowledge. Diverse stakeholder input appeared to spark interest in the reports and stimulated participation. Varied learning styles, data skills, familiarity with research language, professional needs and understanding of the project influenced individual ability to engage. Some roles and established professional relationships emerged as important for facilitating engagement.

### Context

#### Inner context

Lack of time due to high workloads (often linked to staff shortages and acute care needs in communities), and the complex nature of Indigenous PHC delivery, were reported as dominant barriers to engaging PHC teams and managers, particularly by clinicians.

“*It’s not that the reports are complicated, it’s just that there’s a lot in them and we’re really all very busy and we’ve lots of competing demands. But having the summaries is really helpful.*” (Clinician 4)

The extent to which CQI was locally embedded within organisations and teams influenced engagement. In one organisation, interviewees spoke of senior-level commitment to CQI and the associated expectation that staff would participate in the ESP. Several interviewees believed ESP implementation to be a good fit with the existing regional CQI infrastructure.

“*… it could be done through the CQI facilitators, who work through the services, who then work down through their individual clinicians to get that information integrated into what people do in their everyday working environment.*” (Clinician 7)

Organisational restructuring within a large service and management turnover in the Indigenous PHC sector generally were perceived to negatively impact reach and participation, partly because fewer managers had past involvement in the ABCD programme. Management support for the project was important as it helped staff justify the time required for participating and encouraged the convening of groups to appraise and collectively respond to ESP reports.

#### Outer context

The timeliness of ESP implementation for influencing the development of a national CQI strategy for Indigenous PHC was noted, and several spoke of the function of a jurisdiction-level inter-organisational CQI network in promoting ESP engagement. As one interviewee reflected:

“*…that’s where you’ll engage people, when they are away from their services and they’ve got time to think about this stuff*.” (Clinician 5)

Team challenges included updating distribution lists in response to staff turnover and preparing reports suited to the multiple settings and purposes described by interviewees (e.g. strategic planning, CQI training, information shared with community groups).

In summary, a positive wider environment for CQI, programme history and infrastructure support for CQI (including CQI networks) were likely factors supporting participation in local settings characterised by competing work demands and high staff turnover. Leadership and management support was an important enabler or barrier.

Additional file 1 lists the i-PARIHS constructs with illustrative quotes.

## Discussion

This paper analyses the implementation of the ESP project, a large-scale interactive dissemination project, using the i-PARIHS framework. The project achieved wide distribution of ESP reports and sufficient stakeholder engagement to identify priority evidence-practice gaps, barriers/enablers and strategies for improvement across the scope of PHC. Stakeholders used the findings to inform practice, planning, policy and further research. Repeating the interactive dissemination process in different areas of care (e.g. preventive health, maternal care) also established common priority gaps and improvement barriers, providing evidence to inform policy and system-level change for wide-scale improvement in Indigenous PHC.

Various factors appeared to combine for successful implementation. Online dissemination used and extended an existing CQI research/professional network and could target people at different system levels. The data source was perceived as credible and the research goals aligned with stakeholder motivations (e.g. to improve care, to influence policy). Phases of data reporting and feedback captured stakeholder input, producing new knowledge that reflected real-world settings and experiences. Iterative research processes and developmental evaluation enabled ESP reports and processes to be continuously appraised and adapted to support engagement in the research. The research design and process were perceived as novel, robust and aligned with quality improvement principles. Findings were regarded as relevant to improving PHC systems and practice. Facilitation efforts initiated by some stakeholders, particularly CQI facilitators, offset the team’s distant location. They used their knowledge of individuals and teams (e.g. learning styles, skills, professional needs) and their awareness of context (e.g. CQI culture, community setting, competing work demands) when engaging colleagues with the data.

Some of the project’s strengths also presented implementation challenges. It was impossible to accurately measure reach and response rates. Not knowing the full scope of the wider dissemination of ESP reports (e.g. reports were often forwarded to additional colleagues by direct recipients) limited targeting in subsequent project phases and the gathering of data about how stakeholders used the findings. Iterative research cycles supported engagement and produced meaningful evidence for wide system change, but repetitive processes risked disengagement. Reduced stakeholder participation in later ESP cycles may have been due to research fatigue, particularly as some stakeholders received all ESP reports, across several areas of care. Alternatively, higher participation in the early ESP cycles for child health and chronic illness care may have been because these were the most commonly used CQI audit tools, or because these areas of clinical care comprise a large proportion of PHC practitioner workloads compared with other areas such as preventive care. It is possible that each of these factors influenced stakeholder participation. We relied on interested stakeholders to initiate group processes to interpret ESP data, but the scale of the research precluded the research team from directly influencing contextual factors at team and organisation levels.

### Interpretation and comparison with existing literature

Our research team had conceptualised ESP implementation primarily as a process. This was consistent with facilitation processes defined as enabling individuals, groups or teams to work effectively together to achieve a common goal and emphasising shared experiential learning [52]. Facilitators were not included in the project design. Nevertheless, the project was activated through different levels of facilitation. First, we functioned as external facilitators. Despite limited interpersonal contact with stakeholders and the use of online processes to optimise reach, our roles were active rather than passive, partly due to the concurrent developmental evaluation. We responded to feedback and applied our accumulated experiential knowledge of the delivery context, continually refining reports and processes to strengthen implementation. Second, group survey responses required facilitated processes amongst groups and in workplaces. Third, the interactive dissemination process prompted some stakeholders to act as informal project champions, research facilitators, group or outreach facilitators [53]. CQI practitioners, in particular, acted in a facilitative way as linking agents [53]. Together with clinical experts, some managers and team leaders, CQI practitioners spanned boundaries between (1) our team and stakeholders, (2) the ESP project and CQI practice, and (3) different groups of health professionals. These findings reinforce the concept that facilitation is fluid and interactive [13], with facilitation roles taking different forms [53]. They also highlight the pivotal roles of individual, skilled facilitators in successful implementation [13, 53, 54].

While our findings support the i-PARIHS emphasis on inter-related facilitator roles and facilitation processes, they challenge the conventional notion of facilitation as an active process involving practice-based facilitators. The team had only episodic online contact with stakeholders; however, our approach activated the implementation of a large-scale participatory process at a broad system level.

The developmental evaluation provided insights into the ‘black box’ of ESP implementation [55]. Developmental evaluation is recognised as a suitable evaluation approach for novel and complex initiatives due to its innovative niche and complexity perspective [56]. In our project, it helped the team understand how well the theory-based processes worked and what happened between the team reporting the aggregated CQI data and stakeholders’ interpretation and use of findings. Guiding principles of developmental evaluation include the integration and refinement of evaluation processes as part of project implementation [34, 57]. Our adherence to these principles using an embedded evaluator enabled ongoing critical reflection, assessment and alignment of ESP project characteristics with needs of stakeholders in their contextual settings. Use of developmental evaluation had two key benefits in contrast to conventional evaluation approaches. It took the extent of project changes beyond what would be expected through formative evaluation. Further, it achieved real-time adaptation and tailoring to stakeholders and context, in contrast to more traditional process evaluations that usually provide a retrospective explanatory account of findings. In these respects, developmental evaluation functioned as an additional facilitative factor.

### Implications for practice and future research

The ESP’s interactive dissemination process encouraged online sharing and gathered input in a manner resembling ‘crowdsourcing’ [58], thereby making optimal use of limited resources to spread information and engage stakeholders in data interpretation. Other known advantages of external facilitation include a greater likelihood of empowering local facilitation and action, and less likelihood of facilitators being affected by pressures and dynamics within organisations [13]. This played out in the ESP project by stakeholders independently acting as internal facilitators. However, it took time for awareness and understanding of the project to build amongst stakeholders, and high workloads and staff turnover impacted on the continuity of these facilitation efforts. The individuals known to champion the ESP project generally had some history of involvement in the ABCD CQI programme. In future interactive dissemination processes at scale, we would recommend the early establishment of linking roles within regions or large organisations and a targeted communication strategy to establish and maintain rapport with local-level facilitators. Providing people in facilitation/linkage roles with additional resources would affirm and support their roles. A systematic strategy to present the project and engage stakeholders at national or regional forums could identify possible facilitators [59] while expanding reach.

This research helps to address largely unexplored approaches for engaging a range of stakeholders in the co-production of evidence for improving care [60]. Knowledge co-production recognises that research users (typically healthcare clients) bring expertise about context to the solving of complex care problems [61, 62]. The ABCD National Research Partnership established that targeted, collective efforts are required to achieve consistent high-quality care for Indigenous communities [63, 64]. The ESP project drew on the collective knowledge and experience of researchers and healthcare stakeholders (including Indigenous staff) to co-produce findings. Use of the findings was integrated into the phased dissemination process, leading to improvement strategies. This approach to co-production, based on CQI methodology, has potential for use in other settings.

### The utility of the i-PARIHS framework

Limited work to date has applied the i-PARIHS framework as an analytic tool. We found it useful for analysis because, while it reflects common elements of knowledge translation theory more widely (e.g. relating to context, innovation features, individual characteristics and implementation processes), it particularly highlights ‘how’ implementation is activated. This positioning helped make sense of how ‘interactive dissemination’ worked to achieve successful ESP implementation. Examining implementation through an i-PARIHS lens highlighted interactions between the facilitation, innovation, recipient and context constructs. Better understanding of these interactions is helpful for planning future interventions that reflect a systems approach to improving care and responding to Indigenous PHC contexts. These interdependencies made it difficult to analyse them as discrete constructs, but the areas of facilitation focus identified by the developers of i-PARIHS (e.g. degree of fit of the innovation; motivation of recipients) [11] were useful categorising aids. As might be expected, the focus areas given least attention within the ESP design were those reflecting more holistic facilitation practice (e.g. team building, structuring of learning activities). These were the types of activities initiated by CQI practitioners to support local implementation, reinforcing the utility of i-PARIHS as currently represented for planning and evaluating practice-based facilitation. Our study findings and learnings support the utility of the i-PARIHS framework for planning participatory knowledge translation processes at scale. Use of i-PARIHS when designing the ESP project may have guided prospective linkage and other strategies to more actively engage intended recipients in their settings.

We found the i-PARIHS framework appropriate for analysing implementation in an Indigenous healthcare context. It enabled a nuanced understanding of how the project worked in this complex environment. The i-PARIHS framework uses the term ‘recipients’ to describe stakeholders involved in and affected by implementation. We have deliberately used the term ‘recipients’ only when referring directly to the i-PARIHS construct or to recipients of ESP reports because it implies a one-way flow of information from researchers to stakeholders. An expanded definition of this construct may be required to enhance the framework’s acceptability in a research context where empowering Indigenous stakeholders and working in partnership to collaboratively determine priorities and processes are driving principles.

### Strengths and limitations of the study

Repeating the interactive dissemination and evaluation processes using different sets of data provided a series of feedback opportunities to inform this study. To our knowledge, the i-PARIHS framework has not previously been applied to analyse wide-scale engagement in research, nor to a study in Indigenous PHC. Available data indicated strong reach; however, the study could not measure implementation and adoption of findings. Most interviews were conducted in one Australian jurisdiction in which there was an established CQI infrastructure including CQI facilitators; stakeholders in jurisdictions in which CQI is less established may have other experiences of project participation.

## Conclusions

This study applied the i-PARIHS framework to analyse the implementation of a large-scale knowledge translation project in the Australian Indigenous PHC context. Several key learnings emerge.

Key learning 1: The feedback and interpretation processes used to engage healthcare teams with CQI data can be scaled for higher-level system change without intense facilitation efforts. This broadens the notion of facilitation. In our study, successful implementation combined three facilitation processes, namely online processes by an external research team; active facilitation by targeted stakeholders within their teams and organisations; and developmental evaluation to support real-time adaptation and tailoring to stakeholders and context. Together, these processes enabled a dissemination process modelled on CQI principles to be successfully implemented at a health system level.

Key learning 2: The ESP project demonstrated that participatory research is feasible at scale when based on existing networks. In our research context, stakeholder engagement was initiated through an established research network with a history of collaboration and trust. This was a factor in CQI facilitators and other key stakeholders actively facilitating engagement in workplaces. Researchers designing similar interventions should consider this approach and identify and support organisation- or local-level facilitators as part of the research design.

Key learning 3: The i-PARIHS framework has potential for planning and analysing system-level interventions. Its use in this study has enhanced our understanding of factors contributing to the success and limitations of the interactive dissemination process, and of facilitation. It also highlighted the potential of developmental evaluation for strengthening knowledge translation interventions.

Further studies are needed to explore the role and nature of facilitation in system-level projects aiming for engagement, the use of developmental evaluation in knowledge translation research and the use of interactive dissemination processes in other health settings.

## Box 1: The ‘Engaging Stakeholders in Identifying Priority Evidence-Practice Gaps, Barriers and Strategies for Improvement’ (ESP) project (2014–2017)

***Goals:*** (1) Disseminate regionally and nationally aggregated CQI data from Indigenous primary healthcare (PHC) centres; (2) engage healthcare stakeholders in identifying the evidence-practice gaps, barriers and enablers most critical to improving health outcomes in key areas of clinical care and suggesting improvement strategies.

***Data source:*** 60,000 de-identified clinical audits of patient records, 492 systems assessments [25] from 175 PHC centres participating in the ABCD National Research Partnership (2010–2014) across five Australian jurisdictions.

***Targeted stakeholders and settings:*** Policy-makers, managers, health boards, clinicians and researchers in Indigenous community-controlled and government-operated health centres, regional support organisations, government departments and research institutions.

***Knowledge translation process:*** Interactive dissemination.

Methods: ESP reports were distributed by email through the existing ABCD National Research Partnership network and a snowballing recruitment technique was used to invite wider participation. Feedback was obtained through online surveys. A phased reporting and feedback process was used, culminating in a final report.

*Phase 1: Identification of priority evidence–practice gaps.* Preliminary analysis report of aggregated cross-sectional CQI data distributed with linked survey.

*Phase 2: Identification of barriers, enablers and strategies for addressing identified gaps in care.* Report of trend data relevant to the identified priority evidence–practice gaps distributed with a survey about influences on individual behaviours, the health centre, and wider systems and strategies stakeholders would suggest for modifying barriers and strengthening enablers.

*Phase 3: Provision of feedback on draft final report.* Distributed with a survey gathering feedback on the draft overall findings.

The research team analysed survey responses and prepared reports in collaboration with clinical advisors. The process was repeated using CQI data for child health, chronic illness care, maternal, preventive and mental health, and rheumatic heart disease from 2014 to 2017. ESP methods and the theoretical basis are detailed elsewhere [33].

***Evaluation approach:*** Concurrent developmental evaluation (2014–2017) [65].

***Intended outcome:*** Context-relevant evidence to help inform policies and strategies at multiple levels of the health system to achieve wide-scale improvement in care quality [34, 66].

***Outputs:*** 18 phase and final reports, 6 data supplements, key messages in each area of care, journal articles, conference presentations, evidence brief about CQI in Indigenous PHC. Available at: http://www.healthinfonet.ecu.edu.au/

## Box 2: Team facilitation and developmental evaluation processes, responses and adaptations

*Team facilitation processes*

• Project management and administration

• Preparation of documents/tools - report, survey and email templates (all ESP phases)

• Preliminary analysis of recent aggregated data and trend data

• Compiling of distribution lists

• Collaboration with clinical experts

• Linking with CQI facilitators

• Email distribution of reports and ongoing email communication with stakeholders

• Administering surveys to gather stakeholder input

• Analysis of survey data

• Preparation of reports (repeated in key areas of clinical care)

• Development of supporting resources, e.g. evidence brief

• Participation in evaluation

*Developmental evaluation processes*

• Assessing and responding to implementation experiences, stakeholder feedback and patterns in survey responses, interview data

• Adapting and refining aspects of the project to better meet needs

*Responses and adaptations included:*

• Adjusting structure of reports to improve accessibility

• Modifying data presentation (tables to graphs) to support understanding

• Text explanation and audio-visual link of how to interpret graphs

• Statements about the advantages of participation by different professional groups in summaries and emails

• Reducing length of surveys to reduce repetition and completion time

• Extending survey times, sending email reminders to encourage input

• Revising the number and refining content of phase reports (e.g. explaining theory and data trends, using diagrams, adding ‘how to use this report’)

• Merging phases to reduce demands on stakeholder and team time

• Including photos, coloured banners and graphics to highlight emails

• Publishing separate data supplements to reduce report size

• Involving clinical experts to strengthen interpretation and write-up

• Emails directed specifically to CQI practitioners/leaders in the CQI network, in recognition of their key dissemination/facilitation role

• Producing (to broaden reach and usability):

– group facilitation guide

– plain language summaries of all reports

– one-page overview of key findings in each area of clinical care

– key messages for action from findings in each area of care

*CQI* continuous quality improvement, *ESP* Engaging Stakeholders in Identifying Priority Evidence-Practice Gaps, Barriers and Strategies for Improvement

## Box 3 - Successful implementation in the i-PARIHS framework

*SI* = *Fac*^*n*^(*I* + *R* + *C*)

• SI = Successful implementation

o Achievement of agreed implementation/project goals

o Uptake and embedding of the innovation in practice

o Stakeholders are engaged, motivated and ‘own’ the innovation

o Variation related to context is minimised across implementation settings

• Fac*n* = Facilitation

• *I* = Innovation

• *R* = Recipients (individual and collective)

• *C* = Context (inner and outer)

*Source* [13]

## Additional file

Additional file 1: Example stakeholder quotes aligned with i-PARIHS constructs. (PDF 710 kb)

## Abbreviations

ABCD: Audit and Best Practice in Chronic Disease
CQI: continuous quality improvement
ESP: Engaging Stakeholders in Identifying Priority Evidence-Practice Gaps, Barriers and Strategies for Improvement
i-PARIHS: Integrated Promoting Action on Research Implementation in Health Services
PARiHS: Promoting Action on Research Implementation in Health Services
PHC: primary healthcare
