# Supplementary material for: Application of the i-PARIHS framework for enhancing understanding of interactive dissemination to achieve wide-scale improvement in Indigenous primary healthcare
Source: Health Res Policy Syst. 2018 Nov 29;16:117. doi: 10.1186/s12961-018-0392-z (PMC6267798; doi:10.1186/s12961-018-0392-z)
Supplement: Supplementary file 1 — Example stakeholder quotes aligned with i-PARIHS constructs. (PDF 710 kb) [file 12961_2018_392_MOESM1_ESM.pdf]

Supplementary File

Application of the i-PARIHS Framework for enhancing understanding of interactive dissemination to achieve wide-scale improvement in Indigenous primary healthcare

Example stakeholder quotes aligned with i-PARIHS constructs

iPARIHS constructs	Stakeholder quotes
**Facilitation**	*"I like that – 'This is what you've said, we've taken that on board. This is the next step. What can we do about it?' I think that's really powerful to acknowledge the consultation and to reassure people that their voices have been heard."* (Academic 3)
	"It has been necessary for the ESP team to be flexible to accommodate the way our external experts want to have input, and their capacity to have input. … This flexibility means we gain greater engagement with stakeholders overall." (ESP team member)
	"As the succession of the topics has come out, I think we've seen an improvement in the presentation of the data and even in … the issues you are canvassing." (Manager 2)
	"I think the multi-stage process of presenting the evidence and getting the feedback and then presenting the feedback and more evidence and then asking about barriers and enablers - that iterative process of involvement is unique." (Academic 3)
	"… there was room for staff on the ground to be involved. I felt like that's what they were asking, for everybody to have a look at it and 'Are we going in the right direction? What do you think about it?'" (Clinician 8)
	"We just went through and discussed the graphs, to give the feedback. Actually, they were very interested in the whole lot, it was a very good experience. … I would have liked to have gone through that with management." (CQI practitioner 5)
	"There is a lot of work that can be done to enhance data and information to enable better decision-making, so anything I can do that supports that is a good thing – and I do believe in quality improvement activities, … done well they can be really effective tools for enhanced patient outcomes." (Policy officer 3)
	"Collapsing phase 2 and 3 is sensible, because when people think of barriers, they just naturally think of solutions at the same time." (CQI practitioner 4)
	"The ESP has provided another layer of information that's stimulated thinking and discussion, that's brought in knowledge and expertise and experience from a broad group. So, it's really enriched the work that we've done." (CQI practitioner 1)

**Characteristics of innovation**	*"The narrative is one that's of very strong credibility, but it's about the narrative getting through to the people who need to make change and that's where these processes of communication are particularly important."* (Academic 4)
Underlying knowledge sources	*"The ESP Project – I could see what it was building on … it had all the data, all the evidence and then the involvement of stakeholders to prioritise the evidence-practice gaps – that was the key point of difference for me."* (Academic 3)
Clarity	*"I think the survey is set up as clear as it could be. It has got a bit shorter and that's a good thing. I think the language is simple, plain, straightforward. That's all good, but I'm just concerned that we're not reaching the people we need to be… We need to be going where the practitioners are and getting it out to them there."* (Academic 6)
Degree of fit	*"I found it easy to do. … All the information was there, and it was all very connected, without me having to go around looking for stuff."* (Clinician 1)
	"*I thought the surveys were geared for people working in clinics*". (Policy officer 2)
	"It's a bit remote from the clinical interface" (Clinician 7).
	"I think there was a lot of information for people to process – you need a significant 'sound-bite'." (Policy officer 3)
	"I really like the way you structure the study, so you identify the gaps first, then identify the barriers and enablers, and then try to put strategies or actions how to address those barriers." (Policy officer 1).
	"One of the ways to engage some of the groups might be to lead with the key messages and findings and then have that leading back into the final report … that could perhaps be the conduit." (Academic 6)
	"*People will read the main messages, but they are unlikely to get beyond that. … it's beyond most people's capacity to understand them and to have the time to think about them."* (Clinician 6)
Degree of novelty	*"It's respecting those practitioners, valuing what they have. And that's not [often] done well … engaging people and getting them to think about what they're doing and making them feel that they can be part of improving things."* (Clinician 5)
	"I don't know of anybody else doing this consultative process, to see what needs to go in it. All those other reports are written and interpreted for you. You just read them." (Researcher 2)
Relative advantage	"*It is an important avenue to put my thoughts down on paper .... and say [what] needs to change*." (Manager 2)
	"It certainly summarises some of the findings we've identified in relation to CQI and systems failures … Over the years these have been taken up by management - it's very slow, but things are changing from a systems point of view. This project gives us an opportunity to really reflect on 'OK, what have we done?'" (CQI practitioner 2)
Observable results	*"There's always multiple steps. You think that ... people immediately engage and immediately undertake actions and things immediately change. And it doesn't - it tends to be an iterative process."* (Manager 1)
	"On a macro level, it provides this large scale, very hard to argue with, evidence for why action is needed, and why support from the wider health system, government funders is needed, in terms of resource allocation." (Academic 3)
**Recipients**	*"All of my motivation is to improve patient outcomes and I think the ESP project is strategic and policy realm, to include things that are outside what would traditionally be captured in the audit and CQI process*." (Manager 2)

Motivation	*"If I know it is going to be of some benefit and relevant to me, and I can use it, then I'm more inclined to spend the extra amount of time on it*." (CQI practitioner 5)
	"I wanted to know what was happening across the [jurisdiction] in child health. I had a vested interest in that report, because it gave me knowledge that I would otherwise have had to go looking for. I really liked the box-and-whisker plots – I wanted to read your interpretation and see if I got the same interpretation. They took time - even as an epidemiologist it's not something I can pick up and flick through [but] I wanted all that detail." (Academic 2)
	"It made me think outside and my bubble and go, 'Wait a minute. This isn't just happening here.' And look at how other people are dealing with them, and 'Can I transport that to here? What can I change to make that work here?'" (Clinician 1)
Values and beliefs	*"It's CQI, they are using this data. These are people working in chronic disease in remote primary health care, so it's important they have their say, and that they're listened to… and have the chance to have input."* (CQI practitioner 4)
	"I'm invested in it and believing in data driven health improvement and seeing the value of that." (CQI practitioner 3)
	"I've seen the power of data to bring people together and focus them and align their intentions and their practices and strategies. It can be transformative." (Manager 2)
Consensus	*"There was a lot of discussion about concepts, what exactly the questions were asking - there were slight differences of opinion and we're working in different health centres and our experiences are different - and coming up with a consensus about how we would respond*." (Clinician 3)
Local opinion leaders	*"We've had some very senior buy-in. We do have people who've been here for a while, there is somewhat of a culture of CQI. I do wonder how deeply that's invested in, by other practitioners."* (Manager 2)
Skills and knowledge	*"For me, it's important to be able to express what we see are the issues, from our point of view. It's just important to be able to identify what are the real priorities and barriers and things that need improving.* (CQI practitioner 2)
	"I thought, I don't understand them - I'm looking at this graph, I'm going to work it out. And it was quite simple once you took the time about it." (Clinician 4)
	"A plain language summary – even if we are talking to health professionals it might make it more intelligible, much more useful, and really facilitate that process of quality improvement that we're trying to make happen." (Clinician 7)
	"To me that large report is not user friendly for on-the-ground staff, but certainly for the next level up, I think it is." *(*Academic 2)
Existing data sources	*"To do [this process] on a big scale and get good data and information and good recommendations is really, really important for us, who are working on the ground. We can't do it. We just don't have the capacity to do it and we don't have the knowledge*." (Clinician 6)
Time and resources	*"The average practitioner, especially the clinic manager, just doesn't have time. They need a short, pointed report."* (Clinician 1)
	"You could actually go through the survey quite quickly … people in the clinic have the answers, they would know what's going on and what they could and couldn't do [to improve care]." (CQI practitioner 4)

	"I worry that people at the grass roots won't have time to do that long survey, but they can give you some pretty valuable feedback." (Policy officer 2)
	"Barriers to participating are only the time limits, over-commitment with workload… I need to make the time." (Manager 4)
	"I know, from working in clinical practice too, that you're always frantically stressed, and you never get to sit down. So, trying to prioritise this sort of process in that context is really difficult." (Academic 5)
	"*It's not that the reports are complicated, it's just that there's a lot in them and we're really all very busy and we've lots of competing demands. But having the summaries is really helpful."* (Clinician 4)
Collaboration and teamwork	*"I have addressed most of the surveys and sent all of them out …we chase each other up and say to each other, 'Have you sent it out?', and when the reminders come back and if they've extended the survey date, we send it out again."* (CQI practitioner 4)
	"We all had an interest, so we had like an informal discussion … which was good in a way because they raised issues I hadn't thought about." (Clinician 1)
	"Clinicians are likely to generate a lot more ideas and will have a lot more thoughts if engaged in discussion rather than sitting there individually responding to questions." (Manager 1)
Professional boundaries and networks	*"This is a national report and what we are seeing here is everybody's problem, so don't think it's about the job you're doing. Also, if everybody is having a problem we can tackle it together. It's far more powerful if it's across the board."* (Clinician 6)
	"I left so many blanks because I didn't feel I was the right person … these [survey questions] are very much about the perspective of someone on-the-ground. I get feedback about …strategic issues, so I have an idea of some of the barriers at a high level." (Policy officer 2)
	"I think there needs to be more thought about how to engage a broader cross-section of practitioners." (Manager 2)
**Inner context: local** Leadership support	*"Well, I know my managers strongly support it, and have an expectation where we'll be actively involved. It validates spending the time."* (Clinician 9)
	"A lot of [remote PHC practitioners] haven't been around long enough. They're invariably having to learn a new system, having to learn to deal with a high degree of complexity in a cross-cultural setting … dealing with a high rotation of other practitioners in the clinic … The barrier to participation is just the nature of the work we do." (Manager 2)
Mechanisms for embedding change	*"It's a big issue if your health centre manager doesn't value CQI, and then if you don't have other staff who really understand it and you've got a lot of locum staff, [but] … our CQI facilitator, as of three or four years, has constantly given the message to the clinic staff that CQI is really important*." (Clinician 9)
**Inner context: organisational** Organisational priorities Structure	*"The general managers, and managers at every level have to understand it and value it. I will forward this information too, to ask teams to look at this and consider the survey, but if they haven't got a health centre manager who's giving them time to participate, then they won't do it. … I think it needs to be planned that you have time to do it with the team. And the CQI facilitators, it would fit naturally with their role."* (Clinical 9)

Leadership & senior management support	*"If I was going to suggest how this could have more impact it would be about cultivating the new person of the moment, our key policy person … then them emailing their network…. To have the appropriate conversations with people who are decision-makers is clearly going to be a facilitator."* (Academic 4)
Systems and processes	*"When there is someone in the CQI position, who can co-ordinate all of us, pull us together and take time over that, to finalise and get surveys back."* (Clinician 3)
	"Roll of staff through here is a problem – staff changing, things get lost and people get off track. It's busy, there's so much going on." (Clinician 1)
Culture	*"I was reflecting on the ESP project and in a sense, I feel as though we'd possibly been successful because I had very little to do with it. …The CQI people were the key in actually driving engagement, they did a lot of the discussion with other team members and independently ran meetings and sought feedback."* (Manager 1)
History of innovation and change	*"I think the credibility of this process is very strong, and the fact that it supports change – people hearing about the pedigree of this project."* (Academic 4)
Absorptive capacity	*"A lot of people are frightened of data and want someone else to interpret it for them … That's where having actual one-on-one communication – and probably the way it's structured [here] it could be done through the CQI facilitators who work with the services, which then work down through their individual clinic to get that information integrated into what people do in their everyday working environment*." (Clinician 7)
**Outer context** Policy drivers and priorities	*"It could come back at government through its own committee that's focused on CQI rather than through [special interest] health networks. That would be another way for it to gain strength and credence."* (Academic 4)
	"Now that this CQI program is being rolled out nationally, there is an opportunity. Around the country, people are still struggling to get CQI going so there's a real opportunity for you mob, who've got all this data … to present highly relevant information." (Clinician 5)
Inter-organisational networks & relationships	*"At the CQI Collaborative - that's where you'll engage people, when they are away from their services and they've got time to think about this stuff. They're already in that mode and whatever's going on can be built on, and you can inform that process."* (Clinician 5)
	"It's about having the resources to get out there and have a credible person who people know and trust, to talk about it – it is very challenging and difficult." (Academic 6)
	"I think the process of disseminating by email, particularly to key people like the CQI teams and … some of those key public health positions and the CQI steering committee - seemed to work well. We seemed to get good engagement." (Manager 1)
